# Basic Fibroblast Growth Factor Contributes to a Shift in the Angioregulatory Activity of Retinal Glial (Müller) Cells

**DOI:** 10.1371/journal.pone.0068773

**Published:** 2013-07-04

**Authors:** Yousef Yafai, Ianors Iandiev, Johannes Lange, Xiu Mei Yang, Peter Wiedemann, Andreas Bringmann, Wolfram Eichler

**Affiliations:** 1 Department of Ophthalmology and Eye Hospital, Medical Faculty, University of Leipzig, Leipzig, Germany; 2 Norwegian Centre for Movement Disorders, Stavanger University Hospital, Stavanger, Norway; 3 Department of Ophthalmology, Eye Institute of Ophthalmology of Chinese PLA, Xijing Hospital, Fourth Military Medical University, Xi’an, Shaanxi Province, China; Université de Technologie de Compiègne, France

## Abstract

Basic fibroblast growth factor (bFGF) is a pleiotropic cytokine with pro-angiogenic and neurotrophic effects. The angioregulatory role of this molecule may become especially significant in retinal neovascularization, which is a hallmark of a number of ischemic eye diseases. This study was undertaken to reveal expression characteristics of bFGF, produced by retinal glial (Müller) cells, and to determine conditions under which glial bFGF may stimulate the proliferation of retinal microvascular endothelial cells. Immunofluorescence labeling detected bFGF in Müller cells of the rat retina and in acutely isolated Müller cells with bFGF levels, which increased after ischemia-reperfusion in postischemic retinas. In patients with proliferative diabetic retinopathy or myopia, the immunoreactivity of bFGF co-localized to glial fibrillary acidic protein (GFAP)-positive cells in surgically excised retinal tissues. RT-PCR and ELISA analyses indicated that cultured Müller cells produce bFGF, which is elevated under hypoxia or oxidative stress, as well as under stimulation with various growth factors and cytokines, including pro-inflammatory factors. When retinal endothelial cells were cultured in the presence of media from hypoxia (0.2%)-conditioned Müller cells, a distinct picture of endothelial cell proliferation emerged. Media from 24-h cultured Müller cells inhibited proliferation, whereas 72-h conditioned media elicited a stimulatory effect. BFGF-neutralizing antibodies suppressed the enhanced endothelial cell proliferation to a similar extent as anti-VEGF antibodies. Furthermore, phosphorylation of extracellular signal-regulated kinases (ERK−1/−2) in retinal endothelial cells was increased when the cells were cultured in 72-h conditioned media, while neutralizing bFGF attenuated the activation of this signaling pathway. These data provide evidence that retinal (glial) Müller cells are major sources of bFGF in the ischemic retina. Müller cells under physiological conditions or transient hypoxia seem to provide an anti-angiogenic environment, but long-lasting hypoxia causes the release of bFGF, which might significantly co-stimulate neovascularization in the retina.

## Introduction

In addition to cataract and glaucoma, proliferative diabetic retinopathy (PDR), retinopathy of prematurity, and pathological processes related to retinal vein occlusion are the leading causes of low vision and blindness in industrialized countries [1]–[3]. In proliferative ischemic retinopathies, regenerative responses may involve initiation and progression of neovascularization, which in turn is largely governed by the activity of pro-angiogenic factors. Neovascularization is an attempt of the retinal tissue to regenerate the blood supply of ischemic-hypoxic retinal areas; however, vessel growth proceeds in an aberrant fashion and causes secondary damage to the tissue. Vascular endothelial growth factor (VEGF-A, commonly and hereafter referred to as VEGF) is the major pro-angiogenic factor released in the retina under ischemic and inflammatory conditions [4]–[6]. However, it has been shown that the synergistic action of other pro-angiogenic factors may be required for the angiogenic effect of VEGF [7]. In addition to VEGF, heparin-binding growth and inflammatory factors, such as basic fibroblast growth factor (bFGF, also known as FGF−2), platelet-derived growth factor, and tumor necrosis factor (TNF)-α, may promote pathological angiogenesis [8]–[10].

BFGF is a pleiotropic cytokine that, in addition to its pro-angiogenic actions, may elicit further effects on retinal cells. In the retina, bFGF occurs in astrocytes, Müller cells, ganglion cells, and pigment epithelium cells. Furthermore, the cytoplasm of photoreceptor cells contains bFGF after light-induced stress [11]. Ischemic conditions and retinal injury cause a rapid increase of retinal bFGF [12]–[14]. Although bFGF is considered neuroprotective in the retina [15]–[17], it also has detrimental effects, such as stimulation of aberrant vessel growth or induction of proliferation and dedifferentiation of Müller cells [18]. Proliferating Müller cells seem to downregulate the expression of glutamine synthetase, raising the possibility that unregulated glutamate levels lead to enhanced glutamate-mediated neurotoxicity [19]. It has been demonstrated that bFGF induces extracellular matrix proteolysis, as well as proliferation and migration of several micro- and macrovascular endothelial cells [2]–[22]. It has also been shown that bFGF and VEGF act synergistically on microvascular endothelial cells [23], with bFGF effects that are in part mediated by stimulation of a VEGF release from Müller cells and vascular endothelial cells [24], [25].

Although retinal glial cells upregulate VEGF under ischemic-hypoxic conditions [26], [27], the role of Müller cells in promoting retinal neovascularization is not completely understood. There is evidence to suggest that Müller cells exert angiostatic effects under normoxic as well as hypoxic conditions. Thus, Müller cells provide an antiproliferative environment for vascular endothelial cells, mediated by the release of soluble anti-angiogenic factors such as pigment epithelium-derived factor (PEDF), thrombospondin (TSP)−1, prolactin, and transforming growth factor (TGF)-β [2]–[33]. It has been shown, for example, that the expression of TGF-β2 and PEDF is decreased in Müller cells under hypoxic conditions; however, the secretion of TSP−1 increased, and conditioned media from cultured Müller cells inhibit rather than stimulate the proliferation of retinal microvascular endothelial cells [3]–[32]. We investigated whether, and under which conditions, Müller cells promote retinal neovascularization. We also determined the conditions that Müller cells may be induced to secrete bFGF, and further examined whether bFGF is involved in their pro-angiogenic actions. In addition, we used immunohistochemistry to determine the glial localization of bFGF in the ischemic retinal tissues of man and rat.

## Results

### Glial Localization of bFGF in Excised Human Retinal Tissues

**Figure 1:**
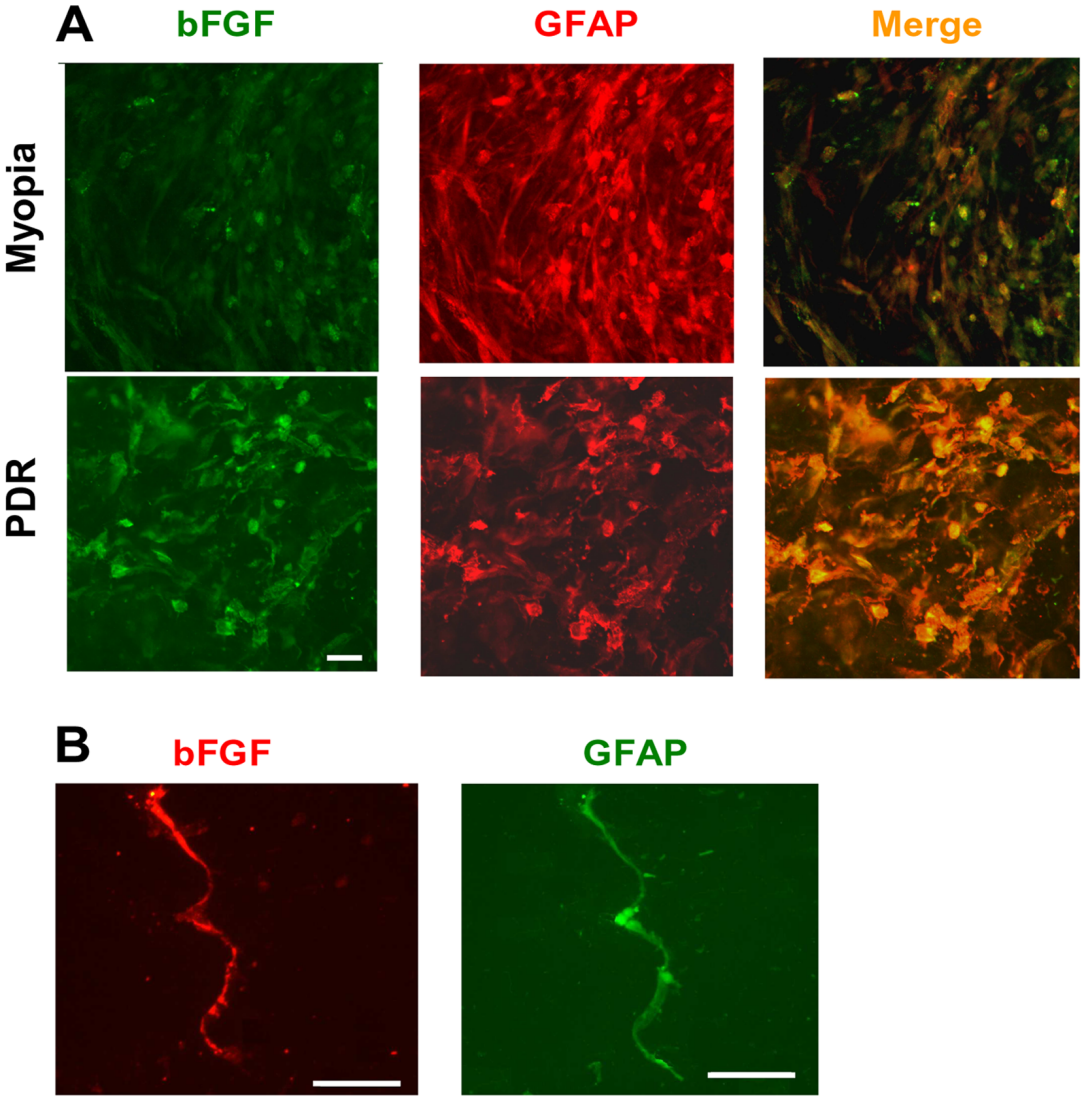
Müller cells express bFGF in retinal tissue. Panel **A** demonstrates glial localization of bFGF (*green* immunostaining) in a surgically excised fibrovascular membrane from a subject with non-hypoxic pathologic myopia and a patient with PDR (scale bar, 25 µm). **B**: Freshly dissociated human Müller cells express bFGF (*red*) and GFAP (*green;* scale bar, 50 µm). Co-staining of GFAP in panel **A** yielded a *yellow* merge signal. Parallel tissue samples or cells stained with nonimmune IgG did not fluoresce (not shown).

To determine whether retinal glial (Müller) cells produce bFGF, we stained slices of surgically excised fibrovascular membranes from patients with ischemic PDR with antibodies specific for bFGF and the glial cell marker GFAP. For comparison, we investigated neovascular membranes from subjects with non-hypoxic pathological myopia. The tissue from myopic eyes demonstrated GFAP-labeled structures that virtually displayed bFGF at a low level, in addition to GFAP-labeled structures devoid of bFGF immunoreactivity. In contrast, almost all GFAP-positive structures in the diabetic fibrovascular membrane displayed bFGF labeling at a higher level (Fig. 1A). To confirm that Müller cells produce bFGF, we isolated single Müller cells acutely from human retinal tissue and subjected them to immunofluorescence labeling. These experiments revealed that isolated retinal glial (Müller) cells displayed immunoreactivity with the bFGF-specific antibody (Fig. 1B). These findings suggest that bFGF immunoreactivity, in PDR fibrovascular membranes coinciding with GFAP labeling, is largely related to bFGF expression by Müller cells.

### Localization of bFGF in the Ischemic Rat Retina

**Figure 2:**
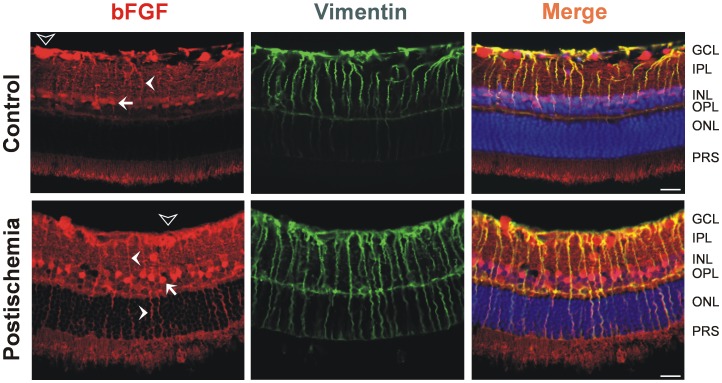
Increased immunostaining of bFGF is a hallmark of ischemia-reperfusion in the rat retina. The images display representative slices of a control retina (*upper panel*) and a retina obtained 3 d after reperfusion (*lower panel*). Specimens were labeled with antibodies against bFGF (*red*) and vimentin (*green*). Co-labeling yielded a *yellow* merge signal, and cell nuclei were labeled with Hoechst 33258 (*blue*, scale bar, 20 µm). Note increasing vimentin and bFGF labeling in the outer nuclear layer (ONL) after ischemia as compared to the control tissue. *Unfilled arrowhead*, ganglion cell soma; *arrow*, Müller cell soma; *filled arrowhead*, Müller cell process. GCL, ganglion cell layer; INL, inner nuclear layer; IPL, inner plexiform layer; OPL, outer plexiform layer; PRS, photoreceptor segments.

Considering a possible glial involvement in retinal bFGF supply, we next investigated whether retinal ischemia-reperfusion injury may lead to altered levels of bFGF derived from Müller cells. Retinae were obtained from eyes which had undergone transient retinal ischemia for 1 h, and ischemic retinal tissue was removed 3 d after reperfusion. Using antibodies against bFGF and vimentin, 2-color labeling of the specimens revealed that in the control tissues, vimentin-labeling of end feet and inner retinal processes of Müller cells, as well as Müller cell somata, closely coincided with bFGF immunoreactivity (Fig. 2, *upper panel*). In addition, photoreceptor segments, a subpopulation of ganglion cell somata, and synaptic structures in the inner plexiform layer were labeled with the anti-bFGF antibody. Most bFGF staining appeared to be confined to cell bodies, suggesting that the bFGF protein largely exists in a cell-associated form in the retina. After retinal ischemia-reperfusion (Fig. 2, *lower panel*), immunostaining of the inner and outer nuclear layers was more intense, although the overall pattern of bFGF labeling in the retina did not change. In the outer nuclear layer, bFGF immunoreactivity was co-localized to vimentin, suggesting that Müller cells accumulate increasing amount of bFGF after transient retinal ischemia.

### Release of bFGF from Müller cells

**Figure 3:**
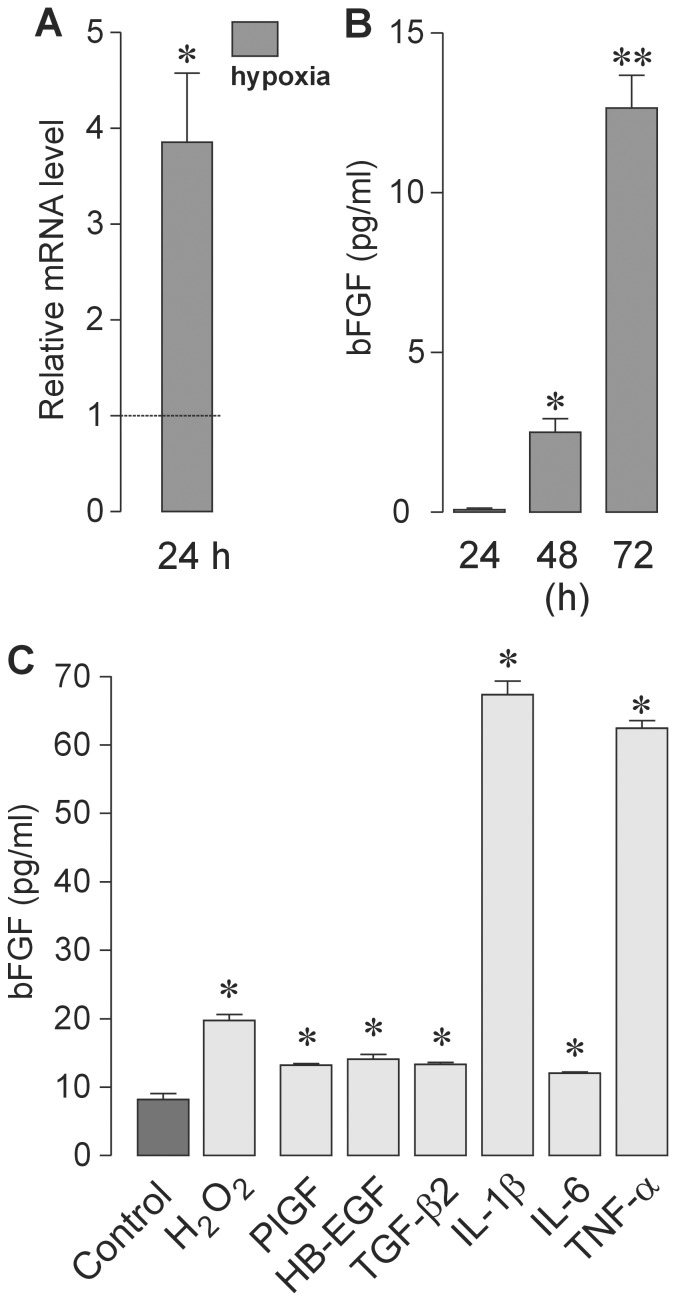
Regulation of bFGF expression by cultured Müller cells. (**A**) Compared to normoxia (*dashed line*), the mRNA level of bFGF in Müller cells under hypoxia (0.2% O_2_; 24 h) was elevated. Changes in bFGF expression were determined using real-time RT-PCR (*n* = 4). (**B**) Glial secretion of bFGF into the culture medium increased with persistence of hypoxia. Müller cells were cultured under hypoxic conditions (0.2% O_2_) for 24, 48, and 72 h, respectively, and the concentration of bFGF in the cultured media was measured using ELISA. (**C**) Oxidative stress induced by addition of H_2_O_2_ (1 mM), as well as various soluble factors, stimulate the secretion of bFGF by Müller cells. The concentration of bFGF in the cultured media was measured using ELISA. The cells were stimulated for 24 h with PlGF−2, HB-EGF, TGF-β2, IL-1β, IL−6, and TNF-α (each at 10 ng/ml). Data are means ± SEM of 3–5 independent experiments. Significant (*P<0.05; **P<0.01) differences *vs*. normoxia (**A**), 24 h (**B**), and control cultures (**C**), respectively, are indicated.

Both acutely isolated and cultured Müller cells were found to express bFGF (cf. Figs. 1B and 3). Interestingly, hypoxic conditions (0.2% O_2_) elicited an increasing expression of bFGF mRNA during 24 h of cell culture (Fig. 3A). Although bFGF was not detectable in supernatants from normoxic 24-h Müller cell cultures at a significant level (data not shown), a release of bFGF was apparent after exposing the cells to hypoxia. However, the bFGF release from Müller cells depends on the duration of hypoxia, as indicated by hypoxic Müller cells cultured for 24 or 72 h, which secreted a greater amount of bFGF (Fig. 3B).

In addition to hypoxia, the secretion of bFGF from Müller cells was stimulated by other factors, including H_2_O_2_-induced oxidative stress and, more importantly, growth factors and cytokines known to be implicated in retinal neovascularization *in situ*, i.e., placenta growth factor (PlGF)-2, heparin-binding epidermal growth factor-like growth factor (HB-EGF), and TGF-β2. However, we found that the pro-inflammatory cytokines, interleukin (IL)-1β and tumor necrosis factor (TNF)-α, exerted the strongest stimulatory effects on bFGF release from Müller cells. In addition, IL−6 stimulated the secretion of bFGF to a moderate extent (Fig. 3C).

### Impact of Müller Cell-derived bFGF on Endothelial Cell Proliferation

**Figure 4:**
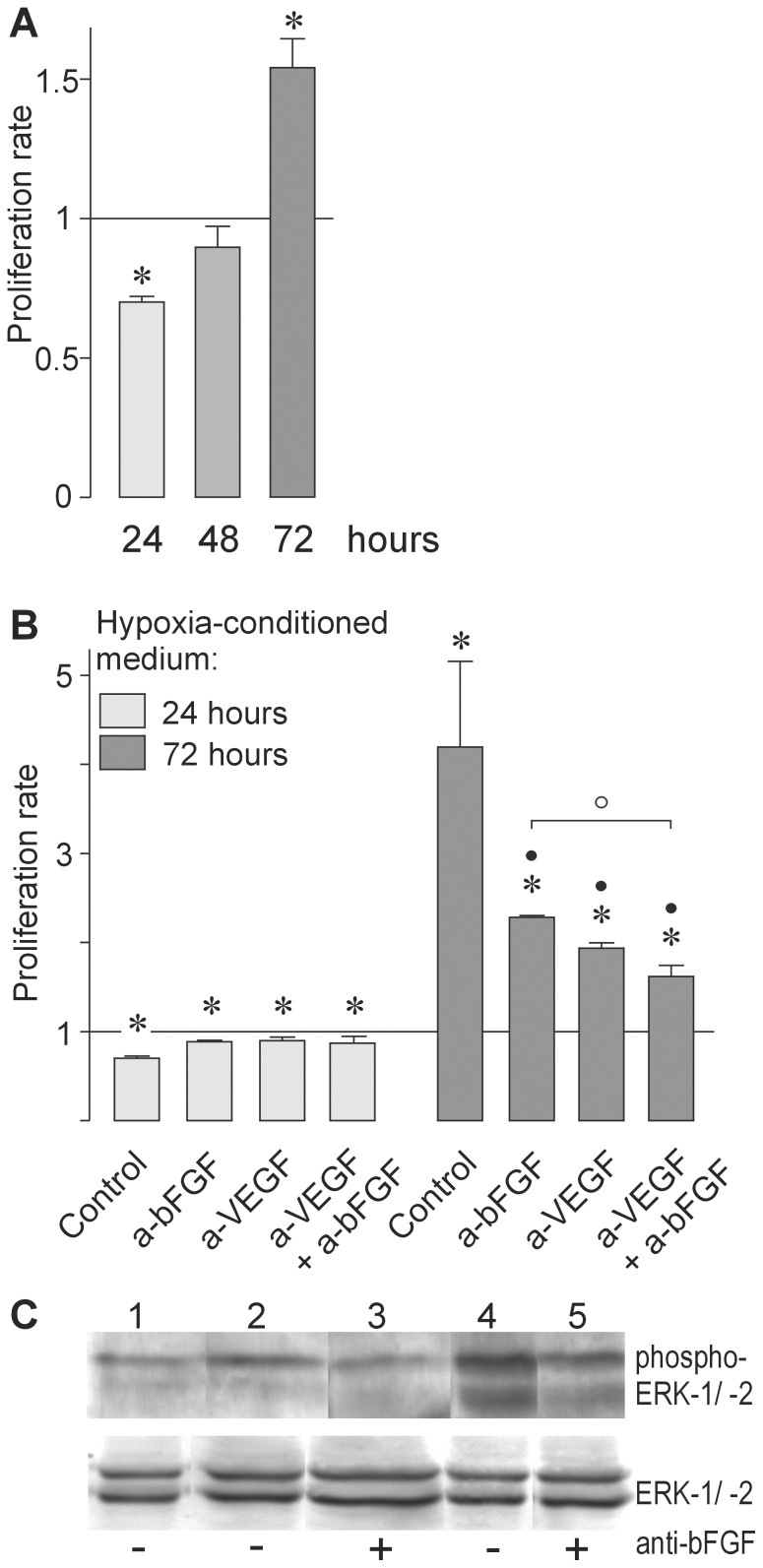
Effects of Müller-cell-derived soluble factors on growth-related activities of retinal microvascular endothelial cells. Hypoxia (0.2% O_2_)-conditioned media from different cultures of Müller cells were employed. Note that proliferation-inducing properties might have varied from one particular medium to another, with different numbers of Müller cells that may have governed a different level of growth factors in these media. (**A**) The proliferation rate of BRECs was determined in the presence of media conditioned for 24, 48, and 72 h under hypoxia. The effects of corresponding media, conditioned under normoxia for 48 and 72 h, were not markedly different from those obtained after 24 h, which caused an approximate 80% reduction of BREC proliferation (*data not shown*). (**B**) and (**C**) Endothelial cell growth-related effects induced by the hypoxia (72 h)-conditioned media was altered in the presence of neutralizing antibodies. (**B**) The proliferation-stimulatory effect of the media on BRECs is significantly attenuated by antibodies directed to bFGF (a-bFGF; 2 µg/ml) or VEGF (a-VEGF; 1 µg/ml), or simultaneous application of both. Significant differences *vs*. hypoxic control cultures maintained in the presence of normal goat immunoglobulin (^•^ *P*<0.05) or cultures in the presence of a-bFGF (^o^ *P*<0.05) are indicated. Data are means ± SEM of 3 independent experiments and are expressed in relative units, i.e., as proportions to basal cellular proliferation in EBM (*P<0.05). (**C**) An increase of ERK-1/−2 phosphorylation is induced by 72-h conditioned media from Müller cells (2,3, normoxia; 3,4, hypoxia) and is reversed by the neutralizing anti-bFGF antibody (+) compared to samples (−), to which normal goat IgG was added. ERK-1/-2 phosphorylation was analyzed by Western blotting.

To elucidate whether Müller cells are able to provide an angiogenesis-permissive microenvironment, we have determined whether conditioned media from Müller cell cultures influence the proliferation of BRECs. As shown in Fig. 4A, the proliferation of BRECs decreased slightly but significantly (*P*<0.05) after transferring hypoxia (0.2% O_2_)-conditioned media, derived from 24-h cultured Müller cells to BRECs. In contrast, supernatants of Müller cells that were maintained for 48 h under hypoxia, did not alter the proliferation rate of BRECs; most notably, media from Müller cells cultured for 72 h under hypoxia significantly (*P*<0.05) stimulated endothelial cellular proliferation (Fig. 4A). Fig. 4B shows that neutralizing anti-bFGF and -VEGF antibodies, to a similar but not complete extent, blocked the proliferation-stimulatory effect of conditioned Müller cell media, suggesting that the pro-angiogenic effect of these media was mainly due to the activity of bFGF and VEGF (also see below). In addition, we have addressed whether Müller-cell conditioned media affect signaling to extracellular signal-regulated kinases (ERK-1/−2)/mitogen-activated protein kinases (MAPK), which serve a critical role in transducing intracellular signals to stimulate proliferation of retinal endothelial cells [34]. We previously demonstrated that exposure of BRECs to 24-h conditioned media can reduce phosphorylation of ERK-1/−2 in response to growth factors [35]. Here, we show an increasing ERK-1/−2 phosphorylation, which was most evident when retinal endothelial cells were cultured in the presence of 72-h conditioned media derived from hypoxic Müller cells (Fig. 4C). As expected, bFGF stimulated phosphorylation of ERK-1/−2 (data not shown). These findings suggest that whereas Müller cells under conditions of short-term hypoxia predominantly secrete anti-angiogenic factors, they release pro-angiogenic factors under conditions of long-term hypoxia, thus stimulating endothelial cell proliferation.

As mentioned previously, the release of bFGF from cultured Müller cells depends on the duration of hypoxia (Fig. 3B). We added blocking antibodies directed against bFGF and VEGF in BREC cultures to determine whether the effect of Müller-cell conditioned media on the proliferation of retinal microvascular endothelial cells were due to increased secretion of bFGF from Müller cells. Neutralizing bFGF or VEGF, or both growth factors, had no significant impact on the proliferation-inhibitory effect of hypoxia-conditioned media derived from 24-h cultured Müller cells. In contrast, the antibodies significantly (*P*<0.05) attenuated the proliferation-stimulatory effect of hypoxia-conditioned media from 72-h cultured Müller cells. Co-administration of anti-bFGF and anti-VEGF antibodies had an even stronger effect when compared to using both antibodies alone (Fig. 4B). Furthermore, we found that a neutralizing anti-bFGF antibody was able to partially reduce ERK-1/−2 phosphorylation in retinal endothelial cells, cultured in the presence of 72-h conditioned media from normoxic or hypoxic Müller cells (Fig. 4C). This indicates that 72-h conditioned media from hypoxic Müller cells mediate their pro-angiogenic activity mainly by bFGF signaling. On the other hand, since neutralization of bFGF and VEGF did not fully prevent the proliferation-stimulatory effect of hypoxia-conditioned Müller cell media, it is likely that hypoxia-exposed Müller cells, in addition to bFGF and VEGF, may release more pro-angiogenic growth factors. Our experiments point to a shift in the angioregulatory capacity of soluble mediators generated by Müller cells under hypoxia, which may be due to elevated bFGF levels.

## Discussion

Retinal neovascularization is considered to be an attempt to reoxygenate hypoxic and ischemic retinal tissue areas. Vasodilatation, inflammation, vascular leakage, glial cell proliferation, neuroprotection, and neurogenesis may accompany tissue repair in the retina. BFGF is an important cytokine that is rapidly released in the retinal tissue under ischemic-hypoxic conditions [14]; it is also involved in various mechanisms of tissue repair and protection including neuroprotection [15]–[17], glial cell proliferation [18], [36], and neovascularization [8], [9]. Retinal glial (Müller) cells are considered to play a crucial role in both neuroprotection and neovascularization, and they are major sources of bFGF [18], [37]. We show that (i) Müller cells synthesize bFGF in both rat and human retinal tissues (Figs. 1 and 2); (ii) bFGF, which was localized to Müller cells, is virtually elevated after transient retinal ischemia-reperfusion (Fig. 2); and (iii) it is retinal glial (Müller) cells that predominantly secrete bFGF in diabetic fibrovascular membranes (Fig. 1).

The role of retinal glial (Müller) cells in the pathogenesis of neovascular retinal disorders, such as PDR, is incompletely understood. On the one hand, Müller cells are considered to be major sources of pro-angiogenic factors in the ischemic-hypoxic retina [11], [2]–[27]. On the other hand, Müller cells may also play an angiostatic role via the release of anti-angiogenic factors such as PEDF, thrombospondin-1, and TGF-β [28]–[32]. In support of this, conditioned media from Müller cells, cultured under normoxic or short-term hypoxic conditions, inhibit rather than stimulate the proliferation of cultured retinal vascular endothelial cells (Refs. 2–32 and Fig. 4A). However, we show that the effect of Müller cells on the proliferation of vascular endothelial cells changes as a function of duration of hypoxia. Indeed, conditioned media from Müller cells exerted angiostatic effects when the cells were grown under short-term hypoxic conditions, but the cells developed a pro-angiogenic activity when cultured under long-term hypoxic conditions (Fig. 4A, B). Under short-term hypoxic conditions, Müller cells released bFGF at low levels (Fig. 3B), and the conditioned media inhibited the proliferation of vascular endothelial cells (Fig. 4A). In contrast, under long-term hypoxic conditions, Müller cells released increasing amounts of bFGF, and the conditioned media stimulated the proliferation of endothelial cells.

Our findings indicated that the stimulatory effect of hypoxia-conditioned Müller cell media on endothelial cell proliferation was partially reversed by a neutralizing anti-bFGF antibody (Fig. 4B). This suggests that glial expression and secretion of bFGF may constitute a major stimulatory event in retinal neovascularization.

The angiostatic effect, mediated by the collective action of Müller-cell derived factors prevailing at an early stage of hypoxia, is to a large extent due to the action of PEDF [35]. The level of PEDF released from Müller cells declines during 24 h of mild hypoxia (2.5% O_2_, Refs. 30,31,38), whereas the PEDF release after 24 h was not significantly altered by strong hypoxia (0.2% O_2_) [38]. The levels of PEDF attained in these experiments were nevertheless adequate to allow endothelial cells to proliferate at a lower rate compared to basal proliferation, virtually independent of the degree of hypoxia [29], [31]. However, increasing levels of proangiogenic growth factors, such as bFGF and VEGF, are capable of overriding this anti-angiogenic activity inherent to PEDF, as we have shown [35]. Thus, an enhanced bFGF/PEDF ratio may also drive the proliferation of endothelial cells in this study, when using conditioned media derived from 72-h cultured hypoxic Müller cells. In these media, PEDF is accumulated at a moderately higher level (approximately 155 pg per 10^5^ cells), when compared to hypoxic media from 24-h cultured Müller cells (approximately 55 pg per 10^5^ cells, data not shown); however, it is unlikely that this difference has a significant impact on endothelial proliferation in the presence of dramatically increasing bFGF levels (Fig. 3B).

Since the ERK-1/-2 MAP kinase signaling pathway controls the proliferation of various cell types, we have determined the state of ERK-1/−2 phosphorylation in retinal endothelial cells. From these experiments, we conclude that the increased proliferative response of endothelial cells is closely paralleled by activation of ERK-1/−2. The response is elicited by soluble Müller cell-derived factors, released under long-term hypoxic conditions. These mediators cause an increase in phosphorylation of threonine 202 and tyrosine 204 on ERK, which is at least in part due to bFGF signaling. Overall, our findings suggest that Müller glial cells, through increased release of bFGF during hypoxia, are significant determinants of a transition from an anti-angiogenic milieu to an angiogenic one, which may allow stimulation of neovascularization *in situ*.

It should be noted that released bFGF is likely to bind to cell surface heparan sulfate proteoglycans, which aid in stimulating bFGF-induced endothelial cell proliferation via high-affinity cell surface tyrosine kinase receptors [39]. This idea complies with the bFGF labeling pattern of the retina in postischemic rats, which indicates that bFGF is largely localized to cell bodies, such as those of the Müllerian glia (Fig. 2). It should also be emphasized in this regard that, besides its pro-angiogenic activity, bFGF has long been known to exert pleiotropic effects on retinal cells with neuro- and gliotrophic actions [36].

We found that bFGF- or VEGF-neutralizing antibodies can block the stimulatory effect of conditioned Müller cell media on endothelial cell proliferation to a similar extent, while the inhibitory effect related to antibody co-administration was even enhanced compared to applying each antibody alone (Fig. 4B). One would have expected additive effects of bFGF and VEGF, if bFGF- and VEGF-induced intracellular signaling cascades are synergistically activated to induce endothelial cell proliferation. It has been shown that the synergistic action of pro-angiogenic factors is required for the angiogenic effect of VEGF [7]. The present results might indicate that bFGF-induced intracellular signaling is activated to support the stimulatory effect of VEGF on the proliferation of retinal vascular endothelial cells. It is also possible that bFGF stimulates the secretion of VEGF from retinal endothelial cells. It has been demonstrated that bFGF stimulates the secretion of VEGF from vascular smooth muscle endothelial and Müller cells [24], [25], and that both cytokines act synergistically on retinal microvascular endothelial cells and pericytes [23].

Finally, there is evidence to suggest that inflammation plays a central role in the pathogenesis of diabetic retinopathy and aberrant retinal neovascularization [40]–[43]. We found that bFGF secretion of Müller cells was strongly stimulated by the pro-inflammatory cytokines, IL-1β and TNF-α (Fig. 3C). It is conceivable that the pro-angiogenic effect of TNF-α might be related, at least in part to the stimulation of bFGF secretion. It has been proposed that the interaction of inflammatory cells with retinal glial cells is critical for the development of neovascular diseases [41]. For example, invading macrophages and activated microglia in an inflammatory retinal microenvironment are able to produce TNF-α, which may stimulate the expression of angiogenic molecules, such as bFGF, by retinal glial cells [42]. Consistent with a significant role of TNF-α in retinal neovascularization, activation of the nuclear transcription factor, NF-κB, has been demonstrated in retinal glial cells surrounding microvessels [43].

In summary, we found that retinal glial (Müller) cells constitute a major source for bFGF in the ischemic retina and diabetic fibrovascular tissue. The secretion of bFGF by Müller cells is stimulated by various pro-angiogenic growth factors and cytokines. While under relatively short-term (transient) hypoxia, the net activity of soluble factors released from Müller cells provides an anti-angiogenic environment for retinal vascular endothelial cells; persistent hypoxia leads to an increasing release of bFGF from Müller cells, which may become increasingly important for shifting the balance between stimulators and inhibitors of angiogenesis in the retina. In this condition, Müller cells are likely to provide a stimulatory environment for the proliferation of retinal vascular endothelial cells. We conclude that bFGF is among the pro-angiogenic factors released from Müller cells, under conditions of long-lasting hypoxia, to play a key role in co-stimulation of abnormal vessel growth in the retina.

## Materials and Methods

Recombinant human growth factors and cytokines were obtained from R&D Systems (Wiesbaden, Germany). The following antibodies were used for immunohistochemistry: rabbit anti-bFGF (1∶200; Santa Cruz, Heidelberg, Germany), mouse monoclonal anti-glial fibrillary acidic protein (GFAP; clone G-A-5, 1∶200; Sigma-Aldrich, Taufkirchen, Germany), mouse monoclonal anti-vimentin (clone V9, 1∶200; Santa Cruz), cyanogen (Cy)3-conjugated goat anti-rabbit IgG (1∶1000; Dianova, Hamburg, Germany), and Cy2-coupled goat anti-mouse IgG (1∶400; Dianova). Neutralizing goat anti-bFGF and goat anti-VEGF-antibodies were from R&D Systems, Wiesbaden, Germany. Appropriate normal control IgG from rabbit, mouse, and goat serum were included. All other reagents used were purchased from Sigma-Aldrich unless stated otherwise.

### Human Material

Human tissue was used in accordance with applicable laws and the Declaration of Helsinki for research. Tissues were used after patients gave written informed consent, according to procedures approved by the ethics committee of the Leipzig University Medical School. A surgically excised fibrovascular membrane from a patient suffering from PDR and retinal tissue from a patient with non-hypoxic pathologic myopia were used for immunohistochemical staining. Freshly dissociated Müller cells were recovered from pieces of retinal tissue using papain- and DNase I-based enzymatic treatment as described earlier [44].

### Retinal Ischemia-reperfusion

All animal experiments were done in accordance with the European Union (EU) Directive 86/609/EEC and approved by local authorities (Faculty of Medicine at the University of Leipzig and Landesdirektion Leipzig). Transient retinal ischemia was induced in one eye of adult Long Evans rats (250−350 g). The other eye remained untreated and served as a control. Anesthesia was induced with intramuscular ketamine (100 mg/kg body weight) and xylazine (5 mg/kg). The anterior chamber of the treated eye was cannulated from the pars plana with a 27-gauge infusion needle, connected to a bag containing normal saline. The intraocular pressure was increased to 160 mm Hg for 60 min by elevating the saline bag. 3 d after reperfusion, the animals were sacrificed with carbon dioxide and the retinas removed.

### Immunohistochemistry

Retinal tissues were fixed in 4% paraformaldehyde for 2 h. After washing with PBS, the tissues were embedded in 3% agarose (w/v, in PBS) and 80-µm thick slices were cut with a vibratome. The slices were incubated in PBS/5% normal goat serum/0.3% Triton X-100 for 2 h at room temperature and exposed to primary antibodies at 4°C overnight. After washing in PBS/1% bovine serum albumin, the secondary antibodies were applied for 2 h at room temperature. Negative controls were included using appropriate preparations of normal rabbit or mouse normal control IgG (not shown). Images were recorded with a confocal laser-scanning microscope (LSM 510 META; Zeiss, Oberkochen, Germany).

### Cell Cultures

Cells of the human Müller cell line MIO-M1 [45] were grown in tissue culture flasks (Greiner, Nürtingen, Germany) at 37°C, 5% CO_2_, 95% air in Dulbecco's modified Eagle's medium (Invitrogen, Karlsruhe, Germany) supplemented with 10% fetal calf serum (FCS) and 100 U/ml penicillin/100 mg/ml streptomycin. Cells were plated at 1×10^5^ per well in 12-well flat-bottom microtiter plates (Greiner), and allowed to attach for 48 h. Cells were growth-arrested in medium without serum for 16 h, briefly washed and either stimulated with growth factors and cytokines for 24 h or exposed to normoxia and hypoxia (0.2% O_2_) for 24, 48, and 72 h, respectively. In a single control experiment, guinea-pig Müller cells were cultured for 24, 48, and 72 h, respectively, to generate hypoxia (0.2% O_2_)-conditioned media. Conditioned media from guinea-pig Müller cell cultures had similar effects on the proliferation of retinal microvascular endothelial cells as conditioned media from MIO-M1 cell cultures (data not shown); we therefore show only data obtained with conditioned media from MIO-M1 cells. Isolation and culture conditions of guinea-pig Müller cells have been described previously [46].

Bovine retinal endothelial cells (BRECs) were isolated and cultured as previously described [29].

Analysis of ERK-1/−2 phosphorylation was carried out using porcine endothelial cells, which were isolated and maintained under the same conditions. Briefly, eyes obtained from a local slaughterhouse were cut circumferentially at the limbus, and the anterior portions were discarded. The neural retina was detached from the retinal pigment epithelium, collected, and washed free from contaminating pigment epithelium or non-retinal tissue using Ca^2+^- and Mg^2+^-free PBS. Retinal tissue was then digested using 1 mg/ml collagenase/dispase (Roche Molecular Biochemicals, Mannheim, Germany) for 45 min at 37°C and subsequently passed through a 20-gauge needle, a 70- and a 40-µm nylon mesh. Isolated cells were routinely cultured in fibronectin-coated tissue culture dishes under 5% CO_2_ at 37°C in endothelial cell growth medium (Lonza, Verviers, Belgium). Subconfluent cultures, which formed typical cobblestone-like monolayer colonies, were passaged using a trypsin-EDTA solution for endothelial cell cultures. More than 90% of the cells in the cultures were confirmed to be vascular endothelial cells using immunocytochemical staining with an antibody against factor-VIII-related antigen (not shown). Cells from passages 2–5 were used for the experiments.

### Proliferation of BRECs

BRECs were assayed for proliferation by determining the incorporation of 5-bromo-2-deoxyuridine (BrdU; Sigma). Briefly, the cells were cultured to ∼50% confluence and serum-starved for 4–8 h prior to setting up the experiment. The cells were incubated for 24 h either in endothelial cell basal medium (EBM, Lonza) or in the presence of conditioned media from MIO-M1 cell cultures, which had been maintained for 24, 48, and 72 h, respectively, under normoxia or hypoxia (0.2% O_2_). BRECs were allowed to incorporate BrdU (final concentration 10 µM) during a time period of 4 h. Finally, the cells were fixed and incubated with a peroxidase-conjugated mouse anti-BrdU antibody. A colorimetric reaction was generated using 3,3', 5,5'-tetramethylbenzidine as a chromogen. The reaction was quenched with H_2_SO_4_ as specified in the protocol of a commercially available cell proliferation kit (Roche). The absorbance of samples was measured at 450 nm in a spectrophotometer. In several experiments, conditioned media were supplemented with FCS (final concentration, 0.2% v/v) and preincubated for 60 min at 37°C with neutralizing goat anti-bFGF (2 µg/ml), goat anti-VEGF (1 µg/ml) or a mixture of both. Incubation with 2 µg/ml normal goat IgG served as a control.

### Analysis of ERK-1/−2 Phosphorylation

Porcine retinal endothelial cells were incubated in the presence of Müller-cell conditioned media for 15 min at 37°C, and total cell lysates were recovered. In brief, the cells were extracted for 30 min in ice-cold lysis buffer (62.5 mM Tris-HCl, pH 7.6, 1 mM EDTA, 150 mM NaCl, 1% Triton X-100, 1 mM phenylmethylsulfonyl fluoride) supplemented with a phosphatase inhibitor mix (Roche Molecular Biochemicals). Lysates were analyzed by Western Blotting using polyclonal phospho-specific anti-p42/p44 MAP kinase antibodies (rabbit anti-phospho-ERK-1/−2, Thr202 and Tyr204) or antibodies directed against ERK-1/−2 (each from New England Biolabs, Frankfurt/Main, Germany; diluted 1∶2,000). Blots were further incubated with goat anti-rabbit IgG conjugated to alkaline phosphatase (Dianova) and developed with a solution of nitroblue tetrazolium (0.5 mg/ml)/5-bromo-4-chlor-3-indolyl phosphate (0.25 mg/ml; both from Sigma).

### Preparation of Total RNA and Real-Time PCR

Total RNA from MIO-M1 cells was isolated using an RNeasy Mini Kit (Qiagen, Hilden, Germany). An appropriate quality of RNA was confirmed by agarose gel electrophoresis, and the A_260_/A_280_ ratio of optical densities was measured using a NanoDrop 1000 device (Peqlab, Erlangen, Germany). After treatment with DNase I (Roche), cDNA was synthesized from 1 µg of total RNA using a RevertAid H Minus First Strand cDNA Synthesis kit (Fermentas, St. Leon-Roth, Germany). Semi-quantitative real-time RT-PCR was performed with the Single-Color Real-Time PCR Detection System (BioRad, Munich, Germany) using the following primer pairs for human bFGF (NM_002006): sense, CAAACTACAACTTCAAGCAG; anti-sense, GAAACACTCGTCTGTAACAC; and beta-actin (5′-ATGGCCACGGCTGCTTCCAGC-3′ and 5′- CATGGTGGTGCCGCCAGACAG-3). A solution containing 1 µl cDNA, specific primers (0.25 µM each) and 10 µl of iQ™ SYBR Green Supermix (BioRad) was used at 20 µl. The following conditions were applied: initial denaturation and enzyme activation (95°C, 3 min); denaturation, amplification and quantification, 45 cycles at 95°C for 30 s, 58°C for 20 s, and 72°C for 45 s; melting curve, 55°C with gradually increased (0.5°C) temperature up to 95°C. Changes in mRNA expression were calculated according to the 2^−ΔΔCt^ method (Ct, cycle threshold), with ΔCt = Ct_bFGF_ − Ct_beta-actin_ and ΔΔCt = ΔCt_treatment_ − ΔCt_control_. Statistical significance was evaluated with a one sample *t* test.

### Enzyme-Linked Immunosorbent Assay

Cell culture media were assayed for bFGF using a sandwich ELISA (R&D Systems). Supernatants (200 µl) were collected from cell culture wells, cleared by centrifugation, and analyzed in duplicate. Statistical significance was evaluated using a Mann-Whitney *U* test.
